# Special Issue: “10th Anniversary of JCM—Recent Diagnostic and Therapeutic Advances in Gastroenterology and Hepatopancreatobiliary Medicine”

**DOI:** 10.3390/jcm11206008

**Published:** 2022-10-12

**Authors:** Takashi Ueda, Hidekazu Suzuki

**Affiliations:** Division of Gastroenterology and Hepatology, Department of Internal Medicine, Tokai University School of Medicine, 143 Shimokasuya, Isehara, Kanagawa 259-1193, Japan

This Special Issue, “10th Anniversary of JCM—Recent Diagnostic and Therapeutic Advances in Gastroenterology and Hepatopancreatobiliary Medicine”, presents five original articles and two review articles.

In this issue, three original articles from Japan are related to the evaluation of the latest endoscopic technology, from which we can see that advanced innovations are being made that are constantly evolving. Among them, the progress of new endoscopic diagnoses using image enhancement technology is remarkable. Nishizawa et al. in our team investigated the effectiveness of texture- and color-enhancement imaging (TXI, Olympus Co. Ltd. Tokyo, Japan) in the imaging of serrated colorectal polyps, including sessile serrated lesions (SSLs) [1]. In their investigation, SSLs were observed using white-light imaging (WLI), TXI, narrow-band imaging (NBI), and chromoendoscopy with and without magnification. We concluded that TXI was significantly superior to WLI but inferior to chromoendoscopy in the imaging of serrated polyps and the sub-analysis of SSLs [1]. In addition to TXI, each endoscope manufacturer introduced new technologies one after another.

Remarkable advances have also been made in treatment instruments for endoscopic submucosal dissection. Fujinami et al. evaluated the utility of the S-O clip during colorectal endoscopic submucosal dissection (ESD) [2]. They compared the time required for endoscopic treatment, the dissection speed, the en bloc resection rate, and the complication rate between the groups. The S-O clip group had a significantly shorter surgery duration, a significantly higher dissection speed, a significantly higher en bloc resection rate (98.8% vs. 80.9%; *p* ≤ 0.001), and a significantly lower perforation rate (1.3% vs. 4.3%) than the non-S-O clip group, especially in cases of lesions in the right colon [2].

On the other hand, insufflation during endoscopy may cause gastrointestinal symptoms. Therefore, the amount of insufflation should be as small as possible. Furthermore, it is thought that these symptoms can be alleviated by replacing ordinary air with CO_2_. Fujisawa et al. reported the effectiveness of CO_2_ insufflation in patients undergoing nasal endoscopy without sedation [3]. According to their study, visual analog scale (VAS) scores for abdominal distension (15.4 vs. 25.5; *p* < 0.001) and distress from flatus (16.0 vs. 28.8; *p* < 0.001) 2 h postprocedure were significantly reduced in the CO_2_ group [3].

Gastric cancer screening programs are a major problem, especially in East Asian countries where gastric cancer deaths are high. In Japan, while endoscopic gastric cancer screening has been initiated nationwide, the incidence of *Helicobacter pylori* infection has decreased, and the number of cases following *H. pylori* eradication has increased. Moreover, the importance of ABC classification (combination of anti-*H. pylori* IgG antibody test with serum pepsinogen test), which reflects *H. pylori* infection status and gastric atrophy before endoscopic screening, is increasingly recognized. Yashima et al. emphasized that considering its cost effectiveness, disseminating the use of endoscopic screening would be advantageous in establishing a new medical examination provision system that conducts examinations at appropriate screening intervals according to the individual’s background and gastric cancer risks [4].

Gastrointestinal bleeding is a disease with a poor prognosis, especially in older adults and in patients with comorbidities; not only might hemostasis affect prognosis, but so might early drug intervention. In this Special Issue, Ting et al. examined whether early tranexamic acid (TXA) administration reduced the risk of mortality in Taiwanese patients with gastrointestinal bleeding [5]. The incidence of mortality significantly decreased during the first and fourth weeks (adjusted HR (aHR): 0.65, 95% CI: 0.56–0.75). A Kaplan–Meier curve revealed a significant decrease in the cumulative incidence of mortality in the early TXA treatment group (log-rank test, *p* < 0.0001). Conversely, thromboembolic events were not significantly associated with early or late TXA treatment (aHR: 1.03, 95% CI: 0.94–1.12). The Kaplan–Meier curve also revealed no significant difference in either venous or arterial events (log-rank test: *p* = 0.3654 and 0.0975, respectively). They concluded that early TXA treatment was associated with a reduced risk of mortality in patients with gastrointestinal bleedig, without an increase in thromboembolic events [5].

For the treatment of ulcerative colitis and Crohn’s disease, many therapeutic options, including biological agents, have been introduced in recent years. Such new therapeutic interventions are expected to have positive effects on the long-term prognosis and quality of life of patients. As you all know, Crohn’s disease (CD) is known to lead to a poor health-related quality of life (HRQoL). Presented in this Special Issue is a systematic review by Aladraj et al., who evaluated the effects of biological agents and small-molecule drugs in improving the HRQoL of patients with moderate-to-severe CD [6]. Among the 16 multicenter, multinational RCTs, 13 studies showed a significant (*p* < 0.05) improvement in the HRQoL of patients with CD, and finally, revealed a substantial improvement in the HRQoL of patients with CD using biological agents and small-molecule drugs [6].

Furthermore, from the field of liver surgery, Katou et al. also presented new findings regarding surgery for liver metastases [7]. They evaluated the outcomes of patients who underwent liver surgery for liver metastasis of non-colorectal and non-neuroendocrine tumors (NCRNNELMs) and colorectal liver metastases (CRLMs) [7]; they analyzed the prognostic factors of overall and recurrence-free survival, and compared survival between the two groups. The 5-year overall survival rates were 38% for NCRNNELM and 55% for CRLM, suggesting that resection for NCRNNELM showed comparable results to resection for CRLM [7].

In this Special Issue, we assemble these seven outstanding articles showing advances in endoscopic technology, the current state of gastric cancer screening programs in Japan, drug interventions for gastrointestinal bleeding, quality of life in patients with Crohn’s disease with recent new biological treatment options, and liver metastasis surgery. We believe that this special feature will bring further progress in the field of Gastroenterology and Hepatopancreatobiliary Medicine.
